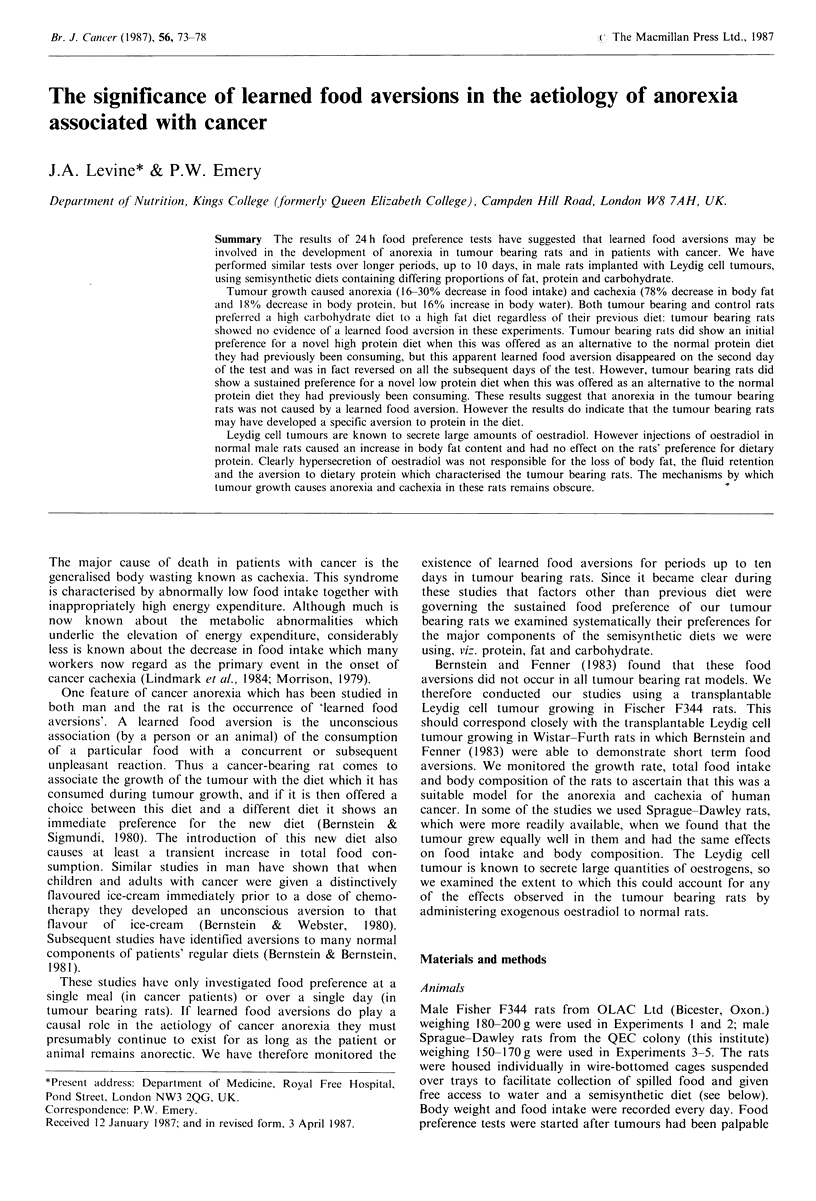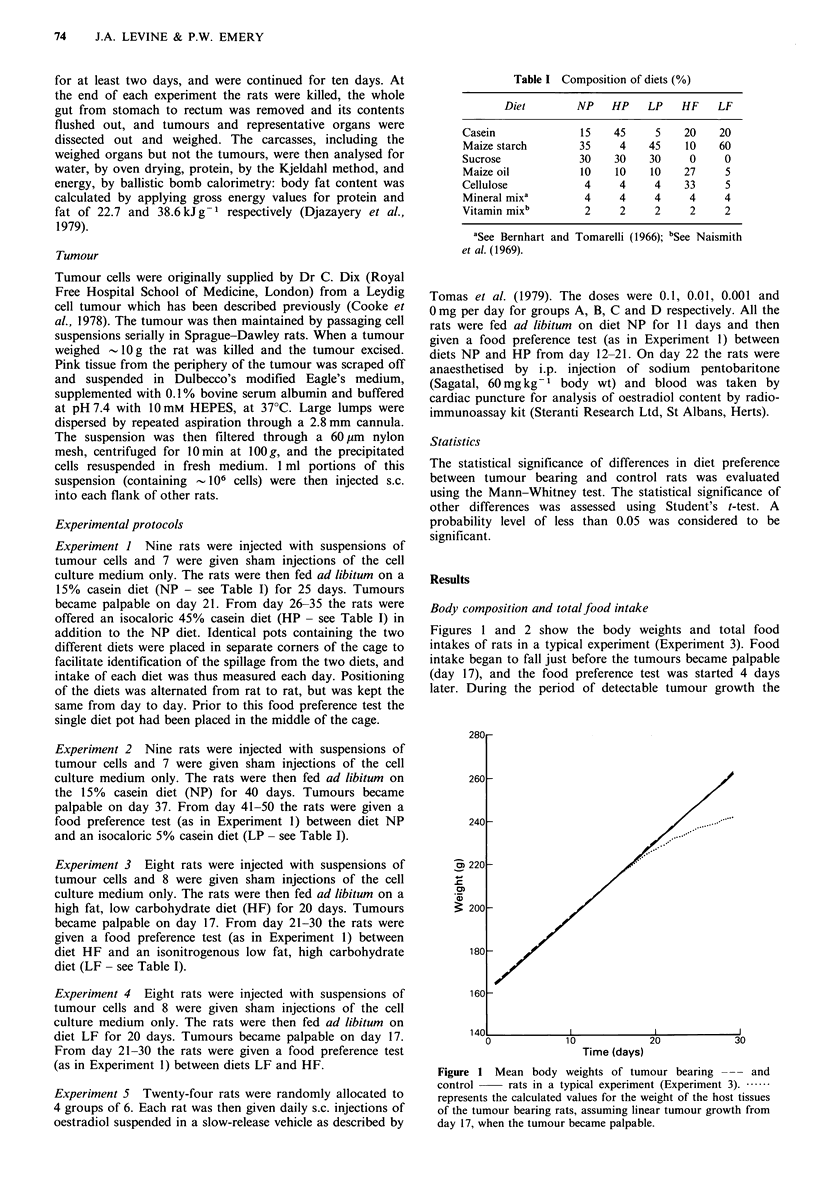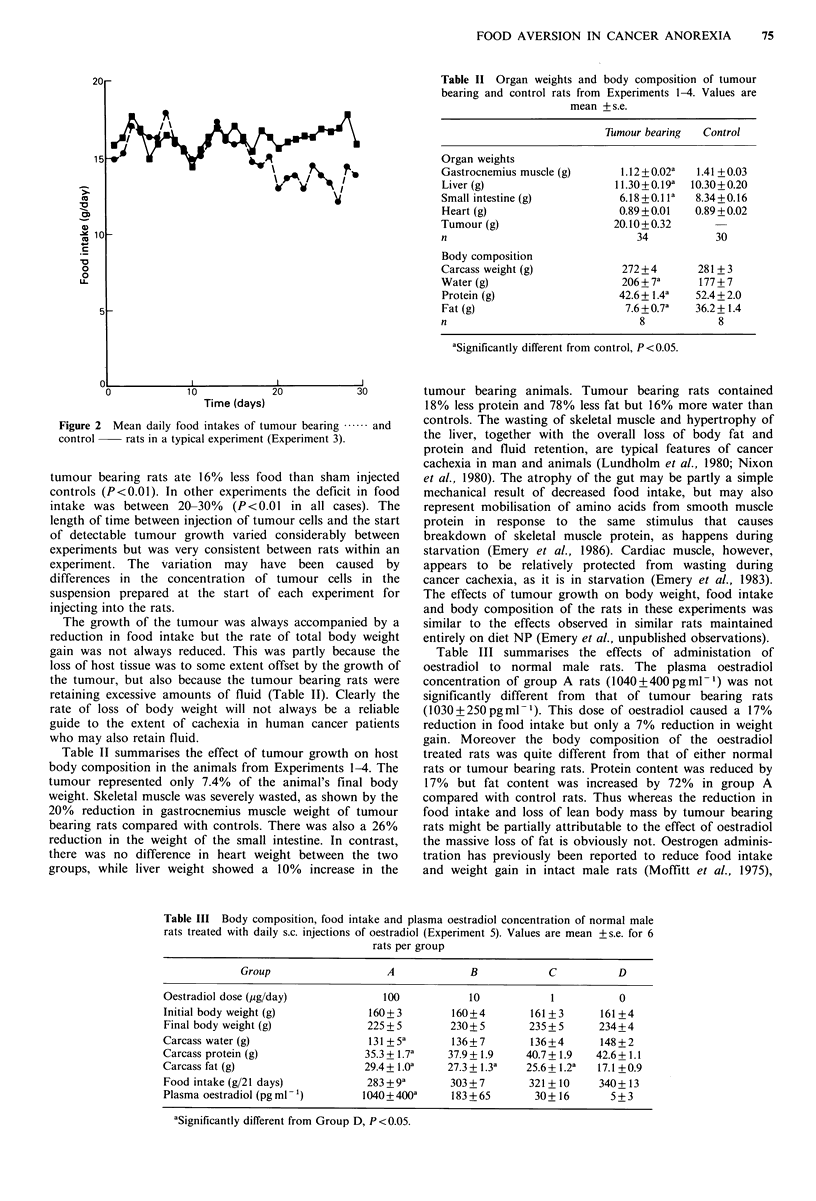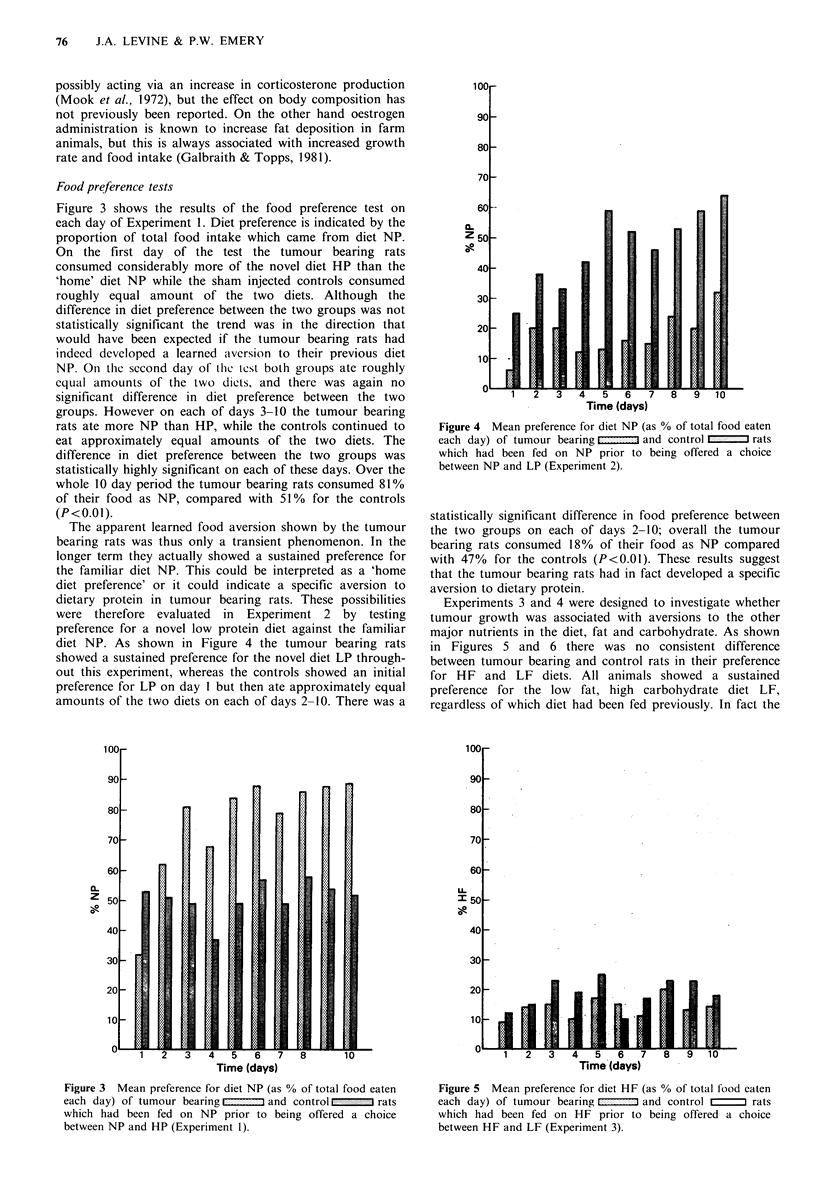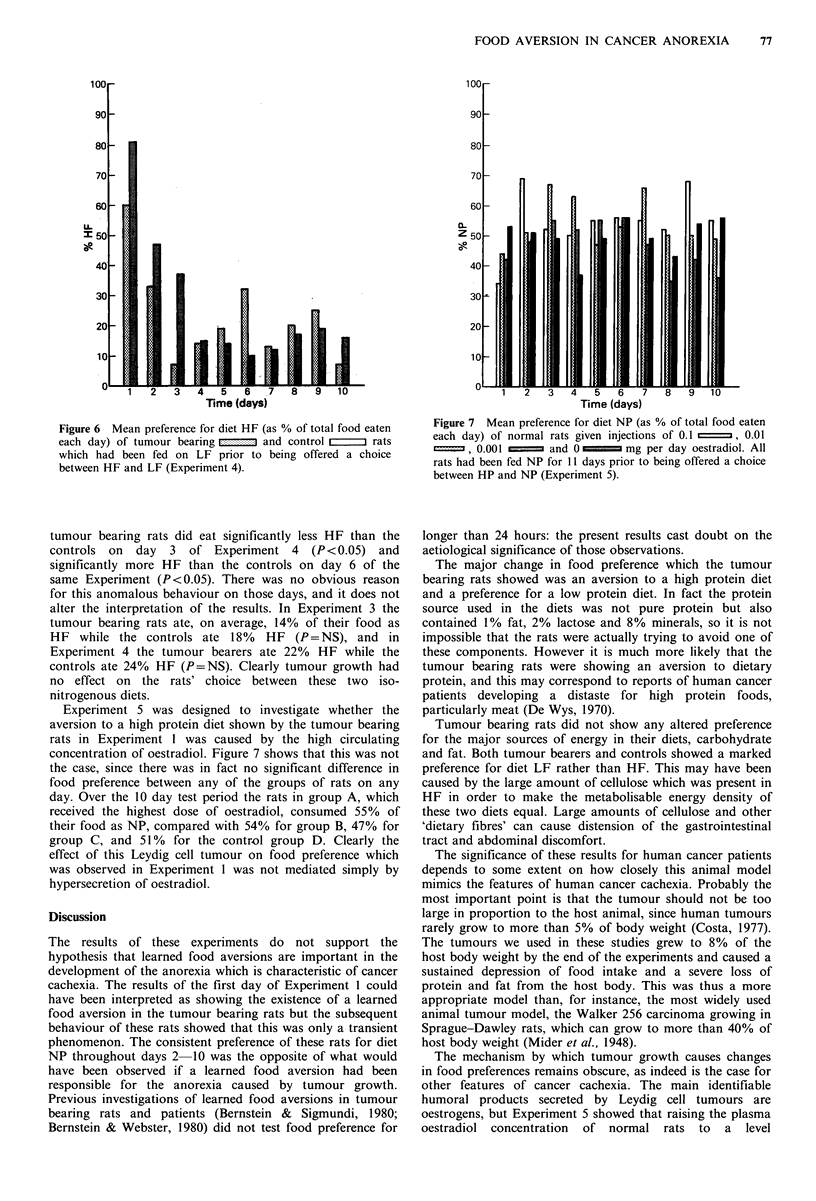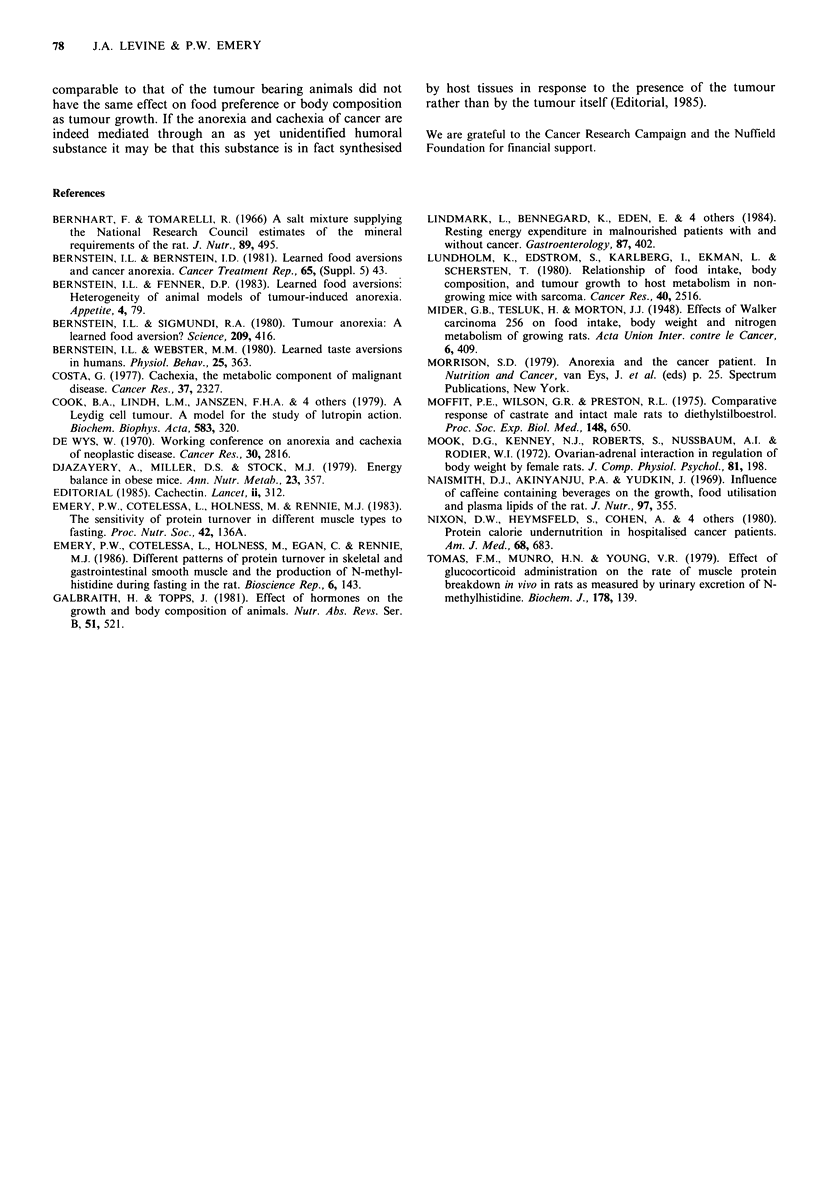# The significance of learned food aversions in the aetiology of anorexia associated with cancer.

**DOI:** 10.1038/bjc.1987.157

**Published:** 1987-07

**Authors:** J. A. Levine, P. W. Emery

## Abstract

The results of 24 h food preference tests have suggested that learned food aversions may be involved in the development of anorexia in tumour bearing rats and in patients with cancer. We have performed similar tests over longer periods, up to 10 days, in male rats implanted with Leydig cell tumours, using semisynthetic diets containing differing proportions of fat, protein and carbohydrate. Tumour growth caused anorexia (16-30% decrease in food intake) and cachexia (78% decrease in body fat and 18% decrease in body protein, but 16% increase in body water). Both tumour bearing and control rats preferred a high carbohydrate diet to a high fat diet regardless of their previous diet: tumour bearing rats showed no evidence of a learned food aversion in these experiments. Tumour bearing rats did show an initial preference for a novel high protein diet when this was offered as an alternative to the normal protein diet they had previously been consuming, but this apparent learned food aversion disappeared on the second day of the test and was in fact reversed on all the subsequent days of the test. However, tumour bearing rats did show a sustained preference for a novel low protein diet when this was offered as an alternative to the normal protein diet they had previously been consuming. These results suggest that anorexia in the tumour bearing rats was not caused by a learned food aversion. However the results do indicate that the tumour bearing rats may have developed a specific aversion to protein in the diet. Leydig cell tumours are known to secrete large amounts of oestradiol. However injections of oestradiol in normal male rats caused an increase in body fat content and had no effect on the rats' preference for dietary protein. Clearly hypersecretion of oestradiol was not responsible for the loss of body fat, the fluid retention and the aversion to dietary protein which characterised the tumour bearing rats. The mechanisms by which tumour growth causes anorexia and cachexia in these rats remains obscure.


Br. J. Cancer (1987), 56, 73–78 © The Macmillan Press Ltd., 1987

### The significance of learned food aversions in the aetiology of anorexia associated with cancer

J.A. Levine* & P.W. Emery

*Department of Nutrition, Kings College (formerly Queen Elizabeth College), Campden Hill Road, London W8 7AH, UK.*

**Summary** The results of 24 h food preference tests have suggested that learned food aversions may be involved in the development of anorexia in tumour bearing rats and in patients with cancer. We have performed similar tests over longer periods, up to 10 days, in male rats implanted with Leydig cell tumours, using semisynthetic diets containing differing proportions of fat, protein and carbohydrate.

Tumour growth caused anorexia (16–30% decrease in food intake) and cachexia (78% decrease in body fat and 18% decrease in body protein, but 16% increase in body water). Both tumour bearing and control rats preferred a high carbohydrate diet to a high fat diet regardless of their previous diet: tumour bearing rats showed no evidence of a learned food aversion in these experiments. Tumour bearing rats did show an initial preference for a novel high protein diet when this was offered as an alternative to the normal protein diet they had previously been consuming, but this apparent learned food aversion disappeared on the second day of the test and was in fact reversed on all the subsequent days of the test. However, tumour bearing rats did show a sustained preference for a novel low protein diet when this was offered as an alternative to the normal protein diet they had previously been consuming. These results suggest that anorexia in the tumour bearing rats was not caused by a learned food aversion. However the results do indicate that the tumour bearing rats may have developed a specific aversion to protein in the diet.

Leydig cell tumours are known to secrete large amounts of oestradiol. However injections of oestradiol in normal male rats caused an increase in body fat content and had no effect on the rats' preference for dietary protein. Clearly hypersecretion of oestradiol was not responsible for the loss of body fat, the fluid retention and the aversion to dietary protein which characterised the tumour bearing rats. The mechanisms by which tumour growth causes anorexia and cachexia in these rats remains obscure.

The major cause of death in patients with cancer is the generalised body wasting known as cachexia. This syndrome is characterised by abnormally low food intake together with inappropriately high energy expenditure. Although much is now known about the metabolic abnormalities which underlie the elevation of energy expenditure, considerably less is known about the decrease in food intake which many workers now regard as the primary event in the onset of cancer cachexia (Lindmark *et al.*, 1984; Morrison, 1979).

One feature of cancer anorexia which has been studied in both man and the rat is the occurrence of 'learned food aversions'. A learned food aversion is the unconscious association (by a person or an animal) of the consumption of a particular food with a concurrent or subsequent unpleasant reaction. Thus a cancer-bearing rat comes to associate the growth of the tumour with the diet which it has consumed during tumour growth, and if it is then offered a choice between this diet and a different diet it shows an immediate preference for the new diet (Bernstein & Sigmundi, 1980). The introduction of this new diet also causes at least a transient increase in total food consumption. Similar studies in man have shown that when children and adults with cancer were given a distinctively flavoured ice-cream immediately prior to a dose of chemotherapy they developed an unconscious aversion to that flavour of ice-cream (Bernstein & Webster, 1980). Subsequent studies have identified aversions to many normal components of patients' regular diets (Bernstein & Bernstein, 1981).

These studies have only investigated food preference at a single meal (in cancer patients) or over a single day (in tumour bearing rats). If learned food aversions do play a causal role in the aetiology of cancer anorexia they must presumably continue to exist for as long as the patient or animal remains anorectic. We have therefore monitored the existence of learned food aversions for periods up to ten days in tumour bearing rats. Since it became clear during these studies that factors other than previous diet were governing the sustained food preference of our tumour bearing rats we examined systematically their preferences for the major components of the semisynthetic diets we were using, *viz.* protein, fat and carbohydrate.

Bernstein and Fenner (1983) found that these food aversions did not occur in all tumour bearing rat models. We therefore conducted our studies using a transplantable Leydig cell tumour growing in Fischer F344 rats. This should correspond closely with the transplantable Leydig cell tumour growing in Wistar–Furth rats in which Bernstein and Fenner (1983) were able to demonstrate short term food aversions. We monitored the growth rate, total food intake and body composition of the rats to ascertain that this was a suitable model for the anorexia and cachexia of human cancer. In some of the studies we used Sprague–Dawley rats, which were more readily available, when we found that the tumour grew equally well in them and had the same effects on food intake and body composition. The Leydig cell tumour is known to secrete large quantities of oestrogens, so we examined the extent to which this could account for any of the effects observed in the tumour bearing rats by administering exogenous oestradiol to normal rats.

### Materials and methods

### *Animals*

Male Fisher F344 rats from OLAC Ltd (Bicester, Oxon.) weighing 180–200 g were used in Experiments 1 and 2; male Sprague–Dawley rats from the QEC colony (this institute) weighing 150–170 g were used in Experiments 3–5. The rats were housed individually in wire-bottomed cages suspended over trays to facilitate collection of spilled food and given free access to water and a semisynthetic diet (see below). Body weight and food intake were recorded every day. Food preference tests were started after tumours had been palpable

*Present address: Department of Medicine, Royal Free Hospital, Pond Street, London NW3 2QG, UK.
Correspondence: P.W. Emery.
Received 12 January 1987; and in revised form, 3 April 1987.

for at least two days, and were continued for ten days. At the end of each experiment the rats were killed, the whole gut from stomach to rectum was removed and its contents flushed out, and tumours and representative organs were dissected out and weighed. The carcasses, including the weighed organs but not the tumours, were then analysed for water, by oven drying, protein, by the Kjeldahl method, and energy, by ballistic bomb calorimetry: body fat content was calculated by applying gross energy values for protein and fat of 22.7 and 38.6 kJ g$^{-1}$ respectively (Djazayery *et al.*, 1979).

*Tumour*

Tumour cells were originally supplied by Dr C. Dix (Royal Free Hospital School of Medicine, London) from a Leydig cell tumour which has been described previously (Cooke *et al.*, 1978). The tumour was then maintained by passaging cell suspensions serially in Sprague–Dawley rats. When a tumour weighed ~10 g the rat was killed and the tumour excised. Pink tissue from the periphery of the tumour was scraped off and suspended in Dulbecco's modified Eagle's medium, supplemented with 0.1% bovine serum albumin and buffered at pH 7.4 with 10 mM HEPES, at 37°C. Large lumps were dispersed by repeated aspiration through a 2.8 mm cannula. The suspension was then filtered through a 60 $\mu$m nylon mesh, centrifuged for 10 min at 100 *g*, and the precipitated cells resuspended in fresh medium. 1 ml portions of this suspension (containing ~10$^6$ cells) were then injected s.c. into each flank of other rats.

*Experimental protocols*

*Experiment 1* Nine rats were injected with suspensions of tumour cells and 7 were given sham injections of the cell culture medium only. The rats were then fed *ad libitum* on a 15% casein diet (NP – see Table I) for 25 days. Tumours became palpable on day 21. From day 26–35 the rats were offered an isocaloric 45% casein diet (HP – see Table I) in addition to the NP diet. Identical pots containing the two different diets were placed in separate corners of the cage to facilitate identification of the spillage from the two diets, and intake of each diet was thus measured each day. Positioning of the diets was alternated from rat to rat, but was kept the same from day to day. Prior to this food preference test the single diet pot had been placed in the middle of the cage.

*Experiment 2* Nine rats were injected with suspensions of tumour cells and 7 were given sham injections of the cell culture medium only. The rats were then fed *ad libitum* on the 15% casein diet (NP) for 40 days. Tumours became palpable on day 37. From day 41–50 the rats were given a food preference test (as in Experiment 1) between diet NP and an isocaloric 5% casein diet (LP – see Table I).

*Experiment 3* Eight rats were injected with suspensions of tumour cells and 8 were given sham injections of the cell culture medium only. The rats were then fed *ad libitum* on a high fat, low carbohydrate diet (HF) for 20 days. Tumours became palpable on day 17. From day 21–30 the rats were given a food preference test (as in Experiment 1) between diet HF and an isonitrogenous low fat, high carbohydrate diet (LF – see Table I).

*Experiment 4* Eight rats were injected with suspensions of tumour cells and 8 were given sham injections of the cell culture medium only. The rats were then fed *ad libitum* on diet LF for 20 days. Tumours became palpable on day 17. From day 21–30 the rats were given a food preference test (as in Experiment 1) between diets LF and HF.

*Experiment 5* Twenty-four rats were randomly allocated to 4 groups of 6. Each rat was then given daily s.c. injections of oestradiol suspended in a slow-release vehicle as described by Tomas *et al.* (1979). The doses were 0.1, 0.01, 0.001 and 0 mg per day for groups A, B, C and D respectively. All the rats were fed *ad libitum* on diet NP for 11 days and then given a food preference test (as in Experiment 1) between diets NP and HP from day 12–21. On day 22 the rats were anaesthetised by i.p. injection of sodium pentobaritone (Sagatal, 60 mg kg$^{-1}$ body wt) and blood was taken by cardiac puncture for analysis of oestradiol content by radio-immunoassay kit (Steranti Research Ltd, St Albans, Herts).

**Table I** Composition of diets (%)

| *Diet* | *NP* | *HP* | *LP* | *HF* | *LF* |
|---|---|---|---|---|---|
| Casein | 15 | 45 | 5 | 20 | 20 |
| Maize starch | 35 | 4 | 45 | 10 | 60 |
| Sucrose | 30 | 30 | 30 | 0 | 0 |
| Maize oil | 10 | 10 | 10 | 27 | 5 |
| Cellulose | 4 | 4 | 4 | 33 | 5 |
| Mineral mix[a] | 4 | 4 | 4 | 4 | 4 |
| Vitamin mix[b] | 2 | 2 | 2 | 2 | 2 |

[a]See Bernhart and Tomarelli (1966); [b]See Naismith *et al.* (1969).

*Statistics*

The statistical significance of differences in diet preference between tumour bearing and control rats was evaluated using the Mann–Whitney test. The statistical significance of other differences was assessed using Student's *t*-test. A probability level of less than 0.05 was considered to be significant.

### Results

*Body composition and total food intake*

Figures 1 and 2 show the body weights and total food intakes of rats in a typical experiment (Experiment 3). Food intake began to fall just before the tumours became palpable (day 17), and the food preference test was started 4 days later. During the period of detectable tumour growth the

**Figure 1** Mean body weights of tumour bearing --- and control —— rats in a typical experiment (Experiment 3). ······ represents the calculated values for the weight of the host tissues of the tumour bearing rats, assuming linear tumour growth from day 17, when the tumour became palpable.

**Figure 2** Mean daily food intakes of tumour bearing ······ and control —— rats in a typical experiment (Experiment 3).

tumour bearing rats ate 16% less food than sham injected controls ($P<0.01$). In other experiments the deficit in food intake was between 20–30% ($P<0.01$ in all cases). The length of time between injection of tumour cells and the start of detectable tumour growth varied considerably between experiments but was very consistent between rats within an experiment. The variation may have been caused by differences in the concentration of tumour cells in the suspension prepared at the start of each experiment for injecting into the rats.

The growth of the tumour was always accompanied by a reduction in food intake but the rate of total body weight gain was not always reduced. This was partly because the loss of host tissue was to some extent offset by the growth of the tumour, but also because the tumour bearing rats were retaining excessive amounts of fluid (Table II). Clearly the rate of loss of body weight will not always be a reliable guide to the extent of cachexia in human cancer patients who may also retain fluid.

Table II summarises the effect of tumour growth on host body composition in the animals from Experiments 1–4. The tumour represented only 7.4% of the animal's final body weight. Skeletal muscle was severely wasted, as shown by the 20% reduction in gastrocnemius muscle weight of tumour bearing rats compared with controls. There was also a 26% reduction in the weight of the small intestine. In contrast, there was no difference in heart weight between the two groups, while liver weight showed a 10% increase in the tumour bearing animals. Tumour bearing rats contained 18% less protein and 78% less fat but 16% more water than controls. The wasting of skeletal muscle and hypertrophy of the liver, together with the overall loss of body fat and protein and fluid retention, are typical features of cancer cachexia in man and animals (Lundholm *et al.*, 1980; Nixon *et al.*, 1980). The atrophy of the gut may be partly a simple mechanical result of decreased food intake, but may also represent mobilisation of amino acids from smooth muscle protein in response to the same stimulus that causes breakdown of skeletal muscle protein, as happens during starvation (Emery *et al.*, 1986). Cardiac muscle, however, appears to be relatively protected from wasting during cancer cachexia, as it is in starvation (Emery *et al.*, 1983). The effects of tumour growth on body weight, food intake and body composition of the rats in these experiments was similar to the effects observed in similar rats maintained entirely on diet NP (Emery *et al.*, unpublished observations).

**Table II** Organ weights and body composition of tumour bearing and control rats from Experiments 1–4. Values are mean ±s.e.

| | *Tumour bearing* | *Control* |
|---|---|---|
| Organ weights | | |
| Gastrocnemius muscle (g) | 1.12±0.02[a] | 1.41±0.03 |
| Liver (g) | 11.30±0.19[a] | 10.30±0.20 |
| Small intestine (g) | 6.18±0.11[a] | 8.34±0.16 |
| Heart (g) | 0.89±0.01 | 0.89±0.02 |
| Tumour (g) | 20.10±0.32 | — |
| *n* | 34 | 30 |
| Body composition | | |
| Carcass weight (g) | 272±4 | 281±3 |
| Water (g) | 206±7[a] | 177±7 |
| Protein (g) | 42.6±1.4[a] | 52.4±2.0 |
| Fat (g) | 7.6±0.7[a] | 36.2±1.4 |
| *n* | 8 | 8 |

[a]Significantly different from control, $P<0.05$.

Table III summarises the effects of administation of oestradiol to normal male rats. The plasma oestradiol concentration of group A rats ($1040\pm400\,pg\,ml^{-1}$) was not significantly different from that of tumour bearing rats ($1030\pm250\,pg\,ml^{-1}$). This dose of oestradiol caused a 17% reduction in food intake but only a 7% reduction in weight gain. Moreover the body composition of the oestradiol treated rats was quite different from that of either normal rats or tumour bearing rats. Protein content was reduced by 17% but fat content was increased by 72% in group A compared with control rats. Thus whereas the reduction in food intake and loss of lean body mass by tumour bearing rats might be partially attributable to the effect of oestradiol the massive loss of fat is obviously not. Oestrogen administration has previously been reported to reduce food intake and weight gain in intact male rats (Moffitt *et al.*, 1975),

**Table III** Body composition, food intake and plasma oestradiol concentration of normal male rats treated with daily s.c. injections of oestradiol (Experiment 5). Values are mean ±s.e. for 6 rats per group

| *Group* | *A* | *B* | *C* | *D* |
|---|---|---|---|---|
| Oestradiol dose (μg/day) | 100 | 10 | 1 | 0 |
| Initial body weight (g) | 160±3 | 160±4 | 161±3 | 161±4 |
| Final body weight (g) | 225±5 | 230±5 | 235±5 | 234±4 |
| Carcass water (g) | 131±5[a] | 136±7 | 136±4 | 148±2 |
| Carcass protein (g) | 35.3±1.7[a] | 37.9±1.9 | 40.7±1.9 | 42.6±1.1 |
| Carcass fat (g) | 29.4±1.0[a] | 27.3±1.3[a] | 25.6±1.2[a] | 17.1±0.9 |
| Food intake (g/21 days) | 283±9[a] | 303±7 | 321±10 | 340±13 |
| Plasma oestradiol (pg ml$^{-1}$) | 1040±400[a] | 183±65 | 30±16 | 5±3 |

[a]Significantly different from Group D, $P<0.05$.

possibly acting via an increase in corticosterone production (Mook *et al.*, 1972), but the effect on body composition has not previously been reported. On the other hand oestrogen administration is known to increase fat deposition in farm animals, but this is always associated with increased growth rate and food intake (Galbraith & Topps, 1981).

*Food preference tests*

Figure 3 shows the results of the food preference test on each day of Experiment 1. Diet preference is indicated by the proportion of total food intake which came from diet NP. On the first day of the test the tumour bearing rats consumed considerably more of the novel diet HP than the 'home' diet NP while the sham injected controls consumed roughly equal amount of the two diets. Although the difference in diet preference between the two groups was not statistically significant the trend was in the direction that would have been expected if the tumour bearing rats had indeed developed a learned aversion to their previous diet NP. On the second day of the test both groups ate roughly equal amounts of the two diets, and there was again no significant difference in diet preference between the two groups. However on each of days 3–10 the tumour bearing rats ate more NP than HP, while the controls continued to eat approximately equal amounts of the two diets. The difference in diet preference between the two groups was statistically highly significant on each of these days. Over the whole 10 day period the tumour bearing rats consumed 81% of their food as NP, compared with 51% for the controls ($P<0.01$).

The apparent learned food aversion shown by the tumour bearing rats was thus only a transient phenomenon. In the longer term they actually showed a sustained preference for the familiar diet NP. This could be interpreted as a 'home diet preference' or it could indicate a specific aversion to dietary protein in tumour bearing rats. These possibilities were therefore evaluated in Experiment 2 by testing preference for a novel low protein diet against the familiar diet NP. As shown in Figure 4 the tumour bearing rats showed a sustained preference for the novel diet LP throughout this experiment, whereas the controls showed an initial preference for LP on day 1 but then ate approximately equal amounts of the two diets on each of days 2–10. There was a statistically significant difference in food preference between the two groups on each of days 2–10; overall the tumour bearing rats consumed 18% of their food as NP compared with 47% for the controls ($P<0.01$). These results suggest that the tumour bearing rats had in fact developed a specific aversion to dietary protein.

**Figure 4** Mean preference for diet NP (as % of total food eaten each day) of tumour bearing [dotted bar] and control [bar] rats which had been fed on NP prior to being offered a choice between NP and LP (Experiment 2).

Experiments 3 and 4 were designed to investigate whether tumour growth was associated with aversions to the other major nutrients in the diet, fat and carbohydrate. As shown in Figures 5 and 6 there was no consistent difference between tumour bearing and control rats in their preference for HF and LF diets. All animals showed a sustained preference for the low fat, high carbohydrate diet LF, regardless of which diet had been fed previously. In fact the

**Figure 3** Mean preference for diet NP (as % of total food eaten each day) of tumour bearing [dotted bar] and control [bar] rats which had been fed on NP prior to being offered a choice between NP and HP (Experiment 1).

**Figure 5** Mean preference for diet HF (as % of total food eaten each day) of tumour bearing [dotted bar] and control [bar] rats which had been fed on HF prior to being offered a choice between HF and LF (Experiment 3).

**Figure 6** Mean preference for diet HF (as % of total food eaten each day) of tumour bearing [stippled bar] and control [open bar] rats which had been fed on LF prior to being offered a choice between HF and LF (Experiment 4).

**Figure 7** Mean preference for diet NP (as % of total food eaten each day) of normal rats given injections of 0.1 [bar], 0.01 [bar], 0.001 [bar] and 0 [bar] mg per day oestradiol. All rats had been fed NP for 11 days prior to being offered a choice between HP and NP (Experiment 5).

tumour bearing rats did eat significantly less HF than the controls on day 3 of Experiment 4 ($P<0.05$) and significantly more HF than the controls on day 6 of the same Experiment ($P<0.05$). There was no obvious reason for this anomalous behaviour on those days, and it does not alter the interpretation of the results. In Experiment 3 the tumour bearing rats ate, on average, 14% of their food as HF while the controls ate 18% HF ($P$=NS), and in Experiment 4 the tumour bearers ate 22% HF while the controls ate 24% HF ($P$=NS). Clearly tumour growth had no effect on the rats' choice between these two iso-nitrogenous diets.

Experiment 5 was designed to investigate whether the aversion to a high protein diet shown by the tumour bearing rats in Experiment 1 was caused by the high circulating concentration of oestradiol. Figure 7 shows that this was not the case, since there was in fact no significant difference in food preference between any of the groups of rats on any day. Over the 10 day test period the rats in group A, which received the highest dose of oestradiol, consumed 55% of their food as NP, compared with 54% for group B, 47% for group C, and 51% for the control group D. Clearly the effect of this Leydig cell tumour on food preference which was observed in Experiment 1 was not mediated simply by hypersecretion of oestradiol.

### Discussion

The results of these experiments do not support the hypothesis that learned food aversions are important in the development of the anorexia which is characteristic of cancer cachexia. The results of the first day of Experiment 1 could have been interpreted as showing the existence of a learned food aversion in the tumour bearing rats but the subsequent behaviour of these rats showed that this was only a transient phenomenon. The consistent preference of these rats for diet NP throughout days 2—10 was the opposite of what would have been observed if a learned food aversion had been responsible for the anorexia caused by tumour growth. Previous investigations of learned food aversions in tumour bearing rats and patients (Bernstein & Sigmundi, 1980; Bernstein & Webster, 1980) did not test food preference for longer than 24 hours: the present results cast doubt on the aetiological significance of those observations.

The major change in food preference which the tumour bearing rats showed was an aversion to a high protein diet and a preference for a low protein diet. In fact the protein source used in the diets was not pure protein but also contained 1% fat, 2% lactose and 8% minerals, so it is not impossible that the rats were actually trying to avoid one of these components. However it is much more likely that the tumour bearing rats were showing an aversion to dietary protein, and this may correspond to reports of human cancer patients developing a distaste for high protein foods, particularly meat (De Wys, 1970).

Tumour bearing rats did not show any altered preference for the major sources of energy in their diets, carbohydrate and fat. Both tumour bearers and controls showed a marked preference for diet LF rather than HF. This may have been caused by the large amount of cellulose which was present in HF in order to make the metabolisable energy density of these two diets equal. Large amounts of cellulose and other 'dietary fibres' can cause distension of the gastrointestinal tract and abdominal discomfort.

The significance of these results for human cancer patients depends to some extent on how closely this animal model mimics the features of human cancer cachexia. Probably the most important point is that the tumour should not be too large in proportion to the host animal, since human tumours rarely grow to more than 5% of body weight (Costa, 1977). The tumours we used in these studies grew to 8% of the host body weight by the end of the experiments and caused a sustained depression of food intake and a severe loss of protein and fat from the host body. This was thus a more appropriate model than, for instance, the most widely used animal tumour model, the Walker 256 carcinoma growing in Sprague–Dawley rats, which can grow to more than 40% of host body weight (Mider *et al.*, 1948).

The mechanism by which tumour growth causes changes in food preferences remains obscure, as indeed is the case for other features of cancer cachexia. The main identifiable humoral products secreted by Leydig cell tumours are oestrogens, but Experiment 5 showed that raising the plasma oestradiol concentration of normal rats to a level comparable to that of the tumour bearing animals did not have the same effect on food preference or body composition as tumour growth. If the anorexia and cachexia of cancer are indeed mediated through an as yet unidentified humoral substance it may be that this substance is in fact synthesised by host tissues in response to the presence of the tumour rather than by the tumour itself (Editorial, 1985).

We are grateful to the Cancer Research Campaign and the Nuffield Foundation for financial support.

**References**

BERNHART, F. & TOMARELLI, R. (1966) A salt mixture supplying the National Research Council estimates of the mineral requirements of the rat. *J. Nutr.*, **89**, 495.

BERNSTEIN, I.L. & BERNSTEIN, I.D. (1981). Learned food aversions and cancer anorexia. *Cancer Treatment Rep.*, **65**, (Suppl. 5) 43.

BERNSTEIN, I.L. & FENNER, D.P. (1983). Learned food aversions: Heterogeneity of animal models of tumour-induced anorexia. *Appetite*, **4**, 79.

BERNSTEIN, I.L. & SIGMUNDI, R.A. (1980). Tumour anorexia: A learned food aversion? *Science*, **209**, 416.

BERNSTEIN, I.L. & WEBSTER, M.M. (1980). Learned taste aversions in humans. *Physiol. Behav.*, **25**, 363.

COSTA, G. (1977). Cachexia, the metabolic component of malignant disease. *Cancer Res.*, **37**, 2327.

COOK, B.A., LINDH, L.M., JANSZEN, F.H.A. & 4 others (1979). A Leydig cell tumour. A model for the study of lutropin action. *Biochem. Biophys. Acta*, **583**, 320.

DE WYS, W. (1970). Working conference on anorexia and cachexia of neoplastic disease. *Cancer Res.*, **30**, 2816.

DJAZAYERY, A., MILLER, D.S. & STOCK, M.J. (1979). Energy balance in obese mice. *Ann. Nutr. Metab.*, **23**, 357.

EDITORIAL (1985). Cachectin. *Lancet*, **ii**, 312.

EMERY, P.W., COTELESSA, L., HOLNESS, M. & RENNIE, M.J. (1983). The sensitivity of protein turnover in different muscle types to fasting. *Proc. Nutr. Soc.*, **42**, 136A.

EMERY, P.W., COTELESSA, L., HOLNESS, M., EGAN, C. & RENNIE, M.J. (1986). Different patterns of protein turnover in skeletal and gastrointestinal smooth muscle and the production of N-methylhistidine during fasting in the rat. *Bioscience Rep.*, **6**, 143.

GALBRAITH, H. & TOPPS, J. (1981). Effect of hormones on the growth and body composition of animals. *Nutr. Abs. Revs.* Ser. B, **51**, 521.

LINDMARK, L., BENNEGARD, K., EDEN, E. & 4 others (1984). Resting energy expenditure in malnourished patients with and without cancer. *Gastroenterology*, **87**, 402.

LUNDHOLM, K., EDSTROM, S., KARLBERG, I., EKMAN, L. & SCHERSTEN, T. (1980). Relationship of food intake, body composition, and tumour growth to host metabolism in non-growing mice with sarcoma. *Cancer Res.*, **40**, 2516.

MIDER, G.B., TESLUK, H. & MORTON, J.J. (1948). Effects of Walker carcinoma 256 on food intake, body weight and nitrogen metabolism of growing rats. *Acta Union Inter. contre le Cancer*, **6**, 409.

MORRISON, S.D. (1979). Anorexia and the cancer patient. In *Nutrition and Cancer*, van Eys, J. *et al.* (eds) p. 25. Spectrum Publications, New York.

MOFFIT, P.E., WILSON, G.R. & PRESTON, R.L. (1975). Comparative response of castrate and intact male rats to diethylstilboestrol. *Proc. Soc. Exp. Biol. Med.*, **148**, 650.

MOOK, D.G., KENNEY, N.J., ROBERTS, S., NUSSBAUM, A.I. & RODIER, W.I. (1972). Ovarian-adrenal interaction in regulation of body weight by female rats. *J. Comp. Physiol. Psychol.*, **81**, 198.

NAISMITH, D.J., AKINYANJU, P.A. & YUDKIN, J. (1969). Influence of caffeine containing beverages on the growth, food utilisation and plasma lipids of the rat. *J. Nutr.*, **97**, 355.

NIXON, D.W., HEYMSFELD, S., COHEN, A. & 4 others (1980). Protein calorie undernutrition in hospitalised cancer patients. *Am. J. Med.*, **68**, 683.

TOMAS, F.M., MUNRO, H.N. & YOUNG, V.R. (1979). Effect of glucocorticoid administration on the rate of muscle protein breakdown *in vivo* in rats as measured by urinary excretion of N-methylhistidine. *Biochem. J.*, **178**, 139.